# Assessment of the Composite Dietary Antioxidant Index and Its Association With Overweight and Obesity Among Adolescents: A Cross-Sectional Study in Mangalagiri, Guntur, Andhra Pradesh

**DOI:** 10.7759/cureus.114582

**Published:** 2026-08-15

**Authors:** K Sakshi, Vignesh D, Veerabathina Kamala, Rajeev Aravindakshan, Arti Gupta

**Affiliations:** 1 Community and Family Medicine, All India Institute of Medical Sciences, Mangalagiri, Mangalagiri, IND

**Keywords:** adolescents, bmi-for-age, cdai, composite dietary antioxidant index, cross-sectional study, dietary antioxidants, india, obesity, overweight, oxidative stress

## Abstract

Background and objectives

Adolescence is a critical window for establishing lifelong dietary and cardiometabolic risk, and low- and middle-income countries increasingly face a double burden of malnutrition. Obesity is characterised by chronic low-grade oxidative stress, and dietary antioxidants may modulate this milieu; the Composite Dietary Antioxidant Index (CDAI) summarises overall antioxidant intake from six nutrients. Data on CDAI among Indian adolescents are sparse. We aimed to estimate the prevalence of overweight and obesity, to estimate the CDAI, and to examine its association with nutritional status among adolescents attending an adolescent health clinic in Guntur, Andhra Pradesh.

Methods

This facility-based cross-sectional study was conducted at the State Adolescent Health Resource Centre, All India Institute of Medical Sciences (AIIMS), Mangalagiri (October 2025-March 2026). A total of 109 adolescents aged 10-19 years were enrolled by systematic sampling. A pre-tested questionnaire captured sociodemographic, physical activity, and substance-use data; dietary intake used a single 24-hour recall. The CDAI was computed as the sum of within-sample standardised (z-score) intakes of vitamins A, C, and E, selenium, zinc, and carotenoids. BMI-for-age z-scores used the WHO 2007 growth reference. Analyses used the Shapiro-Wilk test, Wilson 95% CI, chi-square, Kruskal-Wallis, Mann-Whitney U, Spearman correlation, and binary logistic regression (unadjusted; adjusted for age, sex, and physical activity; and for socioeconomic status), with p < 0.05 considered significant.

Results

Participants had a mean age of 16.0 ± 1.9 years and 62.4% (68/109) were female. The prevalence of thinness was 17.4% (19/109; 95% CI: 11.5%-25.6%), overweight 11.9% (13/109; 95% CI: 7.1%-19.3%), and obesity 12.8% (14/109; 95% CI: 7.8%-20.4%); combined overweight/obesity was 24.8% (27/109; 95% CI: 17.6%-33.6%), coexisting with thinness and reflecting a double burden of malnutrition. The median CDAI was -0.83 (IQR: -2.32 to 1.53), and vitamin A had the highest proportion of zero intakes at 31.2% (34/109). CDAI did not differ across nutritional-status categories (p = 0.723) and was not correlated with BMI-for-age z-score (Spearman ρ = 0.065, p = 0.505); it remained a non-significant predictor of overweight/obesity after adjustment for socioeconomic status (adjusted OR = 1.08, 95% CI: 0.96-1.22, p = 0.181). Carbohydrate intake was higher in overweight/obese than in normal/thin adolescents (p = 0.032), whereas energy, protein, and fat did not differ.

Conclusion

A double burden of malnutrition was evident, with overweight/obesity affecting 24.8% and thinness 17.4% of participants. Diet quality was poor: the median CDAI was below the sample average and 31.2% consumed no vitamin A on the recall day, yet antioxidant intake was not associated with nutritional status. Adolescent nutrition programmes should therefore address under- and overnutrition together: promoting vitamin A- and carotenoid-rich foods rather than supplements; counselling on balanced diets; and routine BMI-for-age screening and nutrition education within existing platforms (Rashtriya Kishor Swasthya Karyakram, mid-day meal, and Anganwadi services), with staple-food fortification to close micronutrient gaps.

## Introduction

Adolescence, defined by the World Health Organization (WHO) as the period between 10 and 19 years of age, is a critical window of physical growth, hormonal change, and behavioural formation. Dietary patterns established during this period track into adulthood and shape long-term nutritional and cardiometabolic risk [1,2]. Andhra Pradesh, like other Indian states, is undergoing a nutrition transition marked by the co-existence of undernutrition and overnutrition, the double burden of malnutrition. Analysis of the Comprehensive National Nutrition Survey (CNNS 2016-18) among 31,941 Indian adolescents (10-19 years) reported thinness in 24.4% (95% CI: 23.5%-25.4%), overweight in 4.8% (95% CI: 4.5%-5.1%), and obesity in 1.1% (95% CI: 0.9%-1.3%) [3]. A recent scoping review of Indian studies reported wide variation, with overweight ranging from 1.25%-35.8% and obesity from 0.3%-24.6%, largely attributable to physical inactivity, energy-dense diets, and higher socioeconomic status [4]; a facility-based study from Haryana reported overweight and obesity of 17.1% and 6.8%, respectively [5]. For context, at the population level, the National Family Health Survey reported that 44.0% of men and 41.2% of women aged 15-49 years (i.e., adults) were overweight or obese, illustrating the adult non-communicable disease (NCD) burden into which this adolescent transition feeds [6].

Obesity is associated with chronic low-grade oxidative stress arising from an imbalance between reactive oxygen species (ROS) generation and endogenous antioxidant defences [7]. Adipose-tissue dysfunction, mitochondrial inefficiency, and inflammation amplify ROS production in obese individuals [8]. Dietary antioxidants, vitamins A, C, and E, selenium, zinc, and carotenoids, are key modulators of this milieu, and adequate intake has been linked to reduced lipid peroxidation and improved metabolic homeostasis [9]. The Composite Dietary Antioxidant Index (CDAI), developed by Wright et al. (2004), integrates the standardised intakes of these six components into a single summary measure of dietary antioxidant status, with higher values indicating greater antioxidant intake relative to the study population [10,11]. In adult cohorts, the CDAI has shown inverse associations with metabolic syndrome, cardiovascular risk, and mortality [12]; however, its application in adolescent populations, particularly in India, remains limited.

Although biochemical assays such as thiobarbituric acid reactive substances (TBARS) directly quantify lipid peroxidation, they were beyond the scope of the present study; dietary antioxidant intake was therefore used as an accessible proxy for antioxidant status [13]. Given the paucity of Indian adolescent data on the interplay between dietary antioxidants and nutritional status, and building on the CNNS finding that overweight is more common in early than late adolescence, this study was designed with the following specific objectives. Descriptive objectives were (i) to estimate the prevalence of thinness, overweight, and obesity using WHO 2007 BMI-for-age z-scores; and (ii) to estimate the CDAI and its distribution by sex and age group. Analytical objective was (iii) to examine the association between the CDAI (and its individual components) and nutritional status, both the four-category classification and the binary overweight/obesity outcome, and to identify sociodemographic and lifestyle correlates of overweight/obesity.

## Materials and methods

Study design and setting

A facility-based cross-sectional analytical study was conducted at the State Adolescent Health Resource Centre, a dedicated adolescent health clinic within All India Institute of Medical Sciences (AIIMS), Mangalagiri, a tertiary-care institution serving the largely peri-urban and rural catchment of Guntur district, Andhra Pradesh, India (AIIMS/MG/IEC/2025-26/421, Dated: 29-09-2025). The study period was conducted from October 2025 to March 2026. Throughout this report, the source population is adolescents attending this Adolescent Health Resource Centre; findings should be interpreted as representative of clinic-attending adolescents rather than the general community.

Eligibility criteria

Inclusion Criteria

The inclusion criteria were adolescents aged 10-19 years attending the State Adolescent Health Resource Centre during the study period, availability of informed written consent from a parent or caregiver and availability of assent from the adolescent.

Exclusion Criteria

The exclusion criteria were 1) severe/acute illness at the time of assessment, 2) known diagnosis of cancer, 3) known HIV-positive status, 4) pregnancy, 5) intellectual disability precluding participation, and 6) current use of medications known to affect appetite, growth, or body composition (e.g., systemic corticosteroids) or a known endocrine disorder. No participant met the medication- or endocrine-related exclusions; had any such cases been identified, they would have been excluded. Chronic-illness status was recorded, and no participant reported a chronic condition expected to materially alter dietary intake or body composition; consequently, no additional adjustment for this variable was required.

Sampling and sample size

Participants were enrolled by systematic sampling of adolescents registering at the centre: after a random start, every second eligible adolescent registering on clinic days was invited until the target sample was reached. The sample size was calculated using n = 4pq/d² with an anticipated adolescent obesity prevalence of 6.8% (Seema et al., 2021) [5], 5% absolute precision, and 95% confidence, yielding a minimum of 100. This calculation was based on the primary descriptive (prevalence) objective; the study was not separately powered for the CDAI correlation/regression component, a limitation addressed quantitatively below and in the Discussion. Of 109 adolescents enrolled with complete sociodemographic and gender information, all 109 had complete dietary and anthropometric data and were retained; no participant was excluded for missing data.

Data collection tools and procedures

A pre-tested, structured, interviewer-administered questionnaire was used, administered in the local language (Telugu) by trained personnel and pilot-tested on 10 adolescents (not included in the analysis) for clarity and flow, with minor wording revisions made (Appendix 1). Socioeconomic status was summarised using a household wealth index constructed by principal components analysis (PCA), following the approach adopted by the National Family Health Survey from its third round onward and by the Demographic and Health Surveys [6]. Variables entered into the PCA comprised housing and amenity characteristics (house type, toilet facility, cooking fuel, drinking-water source, presence of a separate kitchen, house ownership, agricultural land, livestock ownership, and the number of rooms per person) together with binary ownership indicators for durable goods (colour television, refrigerator, telephone, electric fan, pressure cooker, clock, watch, bicycle, scooter, motorcycle, sewing machine, grinder, and items of household furniture); near-universal (>95%) and very rare (<5%) items were excluded to ensure stable estimation. Variables were standardised, and the first principal component was retained as the wealth score, explaining 11.2% of the total variance, consistent with the relative socioeconomic homogeneity of this clinic-based sample, after which households were grouped into wealth tertiles (poorer, middle, richer). Physical activity was captured using items adapted from the WHO Global School-based Student Health Survey (GSHS) format [14], recording the number of days per week with ≥60 minutes of activity, and was summarised as low, medium, or high. Tobacco and alcohol use were recorded to characterize the sample; because reported use was negligible (one tobacco user, no alcohol users), these variables were not entered into multivariable models.

Dietary intake was assessed using a single, standardised 24-hour dietary recall. Portion sizes were estimated by the adolescent with the interviewer using standardised household measures and food models. Recalls were processed in DietCal software version 15.1.3 (Profound Tech Solutions, New Delhi, India), which draws on Indian food-composition data, to compute total energy (kcal), carbohydrate (g), protein (g), fat (g), and intakes of vitamin A (µg), vitamin C (mg), vitamin E (mg), selenium (µg), zinc (mg), and carotenoids (µg).

Anthropometry and nutritional-status classification

Weight was measured to the nearest 0.1 kg with a SECA 813 digital scale (seca GmbH & Co. KG, Hamburg, Germany) and height to the nearest 0.1 cm with a SECA 213 stadiometer, using standardised technique by trained personnel with periodic calibration; BMI was calculated as weight (kg)/height² (m²). BMI-for-age z-scores were computed using the WHO 2007 growth reference (Lambda-Mu-Sigma (LMS) method) for school-aged children and adolescents (5-19 years) [15]. Nutritional status was classified as thinness (z < -2 SD), normal (z = -2 to +1 SD), overweight (z > +1 to +2 SD), and obese (z > +2 SD); at 19 years, the +1 SD and +2 SD thresholds correspond to the adult BMI cut-offs of 25.0 and 30.0 kg/m², respectively [16].

Calculation of the Composite Dietary Antioxidant Index

The CDAI was calculated following Wright et al. (2004) [10,11] as CDAI = Σ [(xᵢ - x̄ᵢ)/SDᵢ], where xᵢ is the individual intake of each of the six antioxidant components, and x̄ᵢ and SDᵢ are the corresponding sample mean and standard deviation. By construction, the CDAI has a sample mean of zero and expresses an individual's antioxidant intake relative to the study population.

Statistical analysis

Analyses used Python 3 (pandas, SciPy, statsmodels; Python Software Foundation, Beaverton, Oregon, USA). Normality was assessed with the Shapiro-Wilk test. Continuous variables are summarised as mean ± SD or median (IQR) as appropriate, and categorical variables as frequencies with Wilson score 95% CIs. Because all antioxidant and CDAI variables were non-normally distributed, group comparisons used the Kruskal-Wallis test (≥3 groups) and the Mann-Whitney U test (two groups). Associations between categorical variables used the chi-square test, with each test explicitly matched to its comparison (sex or age group against the four-category status, and against the binary overweight/obesity outcome). Spearman rank correlation assessed associations of the CDAI and its components with BMI-for-age z-score. Binary logistic regression modelled overweight/obesity with the CDAI as the exposure: Model 1 (unadjusted); Model 2 (adjusted for age, sex, and physical activity); and Model 3, which additionally adjusted for socioeconomic status, operationalised as father's education level and the PCA-derived household wealth index (in tertiles). Socioeconomic status was pre-specified as a plausible confounder of both food access and adiposity. Multicollinearity was assessed using variance inflation factors (VIF). Results are reported as odds ratios (ORs) with 95% CIs, and effects are expressed per 1-unit increase in the continuous CDAI. A post hoc analysis of the minimum detectable effect size was performed for the correlation and regression components. A p-value < 0.05 was considered significant.

## Results

Sociodemographic characteristics

During the study period, 109 adolescents were enrolled and provided complete sociodemographic, dietary, and anthropometric data; no records were excluded for missing data, yielding a final analytic sample of 109. The mean age was 16.0 ± 1.9 years (range: 10.0-19.6 years). Most participants were female (68/109; 62.4%, 95% CI: 53.0%-70.9%), and late adolescents (15-19 years) predominated (76/109; 69.7%). Most fathers were engaged in unskilled or daily-labour occupations (57.7%), and 20.8% of both fathers and mothers were illiterate. Based on the PCA-derived household wealth index, participants were distributed across tertiles as poorer (36/109; 33.0%), middle (36/109; 33.0%), and richer (37/109; 33.9%). Only one participant (0.9%) reported tobacco use, and none reported alcohol use. Physical activity was medium in 61.5% and low in 28.4%. Sociodemographic details, including maternal education and the wealth index, are presented in Table 1.

**Table 1:** Sociodemographic characteristics of study participants (n = 109). Percentages for parental education and occupation are of valid responses (education n = 106; occupation n = 104). *Wealth index derived by principal components analysis of household assets and amenities (first component, 11.2% of variance), grouped into study-sample tertiles. PCA = principal components analysis.

Variable	Category	n	%
Gender	Female	68	62.4
	Male	41	37.6
Age group	Early adolescence (10-14 y)	33	30.3
	Late adolescence (15-19 y)	76	69.7
Physical activity	Low	31	28.4
	Medium	67	61.5
	High	11	10.1
Tobacco use	Yes	1	0.9
Alcohol use	Yes	0	0.0
Father's education	Illiterate	22	20.8
	Primary/Middle school	39	36.8
	High school/Intermediate	31	29.2
	Graduate and above	14	13.2
Mother's education	Illiterate	22	20.8
	Primary/Middle school	42	39.6
	High school/Intermediate	32	30.2
	Graduate and above	10	9.4
Father's occupation	Unemployed/Farmer	15	14.4
	Unskilled/Daily labour	60	57.7
	Semi-skilled/Skilled	22	21.2
	Semi-professional and above	7	6.7
Household wealth index (PCA)*	Poorer tertile	36	33.0
	Middle tertile	36	33.0
	Richer tertile	37	33.9

Anthropometric profile and dietary antioxidant intake

The mean BMI was 20.57 ± 4.81 kg/m² (median: 19.26 kg/m²), and the mean BMI-for-age z-score was -0.19 ± 1.71, indicating a distribution marginally below the WHO 2007 reference median. The median CDAI was -0.83 (IQR: -2.32 to 1.53). Vitamin A had the highest proportion of zero intakes (31.2%), consistent with low consumption of green leafy vegetables, liver, and dairy in lower-income households; median selenium intake (22.16 µg/day) was below the ICMR recommended dietary allowance of 40 µg/day for adolescents. Mean total energy intake was 1291.6 ± 363.1 kcal/day, with a median carbohydrate intake of 177.3 g/day (IQR: 149.1-206.9), protein intake of 35.5 g/day (IQR: 26.9-48.8), and mean fat intake of 42.3 ± 15.1 g/day. Full anthropometric, macronutrient, and antioxidant summaries are shown in Table 2.

**Table 2:** Anthropometric characteristics, CDAI, and antioxidant components (n = 109). *Shapiro-Wilk p < 0.05. Antioxidant intakes from a single 24-hour dietary recall. CDAI = Composite Dietary Antioxidant Index.

Variable	N	Mean ± SD	Median	IQR (Q1 to Q3)	Distribution
Weight (kg)	109	48.89 ± 11.98	47.60	40.5 to 54.1	Non-normal*
Height (cm)	109	154.18 ± 9.09	153.00	148.0 to 160.0	Normal
BMI (kg/m²)	109	20.57 ± 4.81	19.26	17.26 to 22.5	Non-normal*
BMI-for-age z-score	109	-0.19 ± 1.71	-0.23	-1.49 to 0.97	Normal
CDAI score	109	0.00 ± 3.72	-0.83	-2.32 to 1.53	Non-normal*
Energy (kcal)	109	1,291.6 ± 363.1	1242.8	1,046.7 to 1,501.3	Normal
Carbohydrate (g)	109	184.1 ± 52.1	177.3	149.1 to 206.9	Non-normal*
Protein (g)	109	39.4 ± 17.7	35.5	26.9 to 48.8	Non-normal*
Fat (g)	109	42.3 ± 15.1	41.9	31.6 to 53.9	Normal
Vitamin A (µg)	109	87.80 ± 334.73	15.20	0.0 to 86.84	Non-normal*
Vitamin C (mg)	109	28.97 ± 26.22	22.74	14.56 to 33.43	Non-normal*
Vitamin E (mg)	109	1.37 ± 1.44	0.93	0.58 to 1.64	Non-normal*
Selenium (µg)	109	31.71 ± 31.77	22.16	10.64 to 41.3	Non-normal*
Zinc (mg)	109	4.76 ± 2.16	4.49	3.4 to 5.77	Non-normal*
Carotenoids (µg)	109	4,691.23 ± 3,513.34	4013.77	2,535.3 to 5,423.1	Non-normal*

Prevalence of thinness, overweight, and obesity

**Figure 1:**
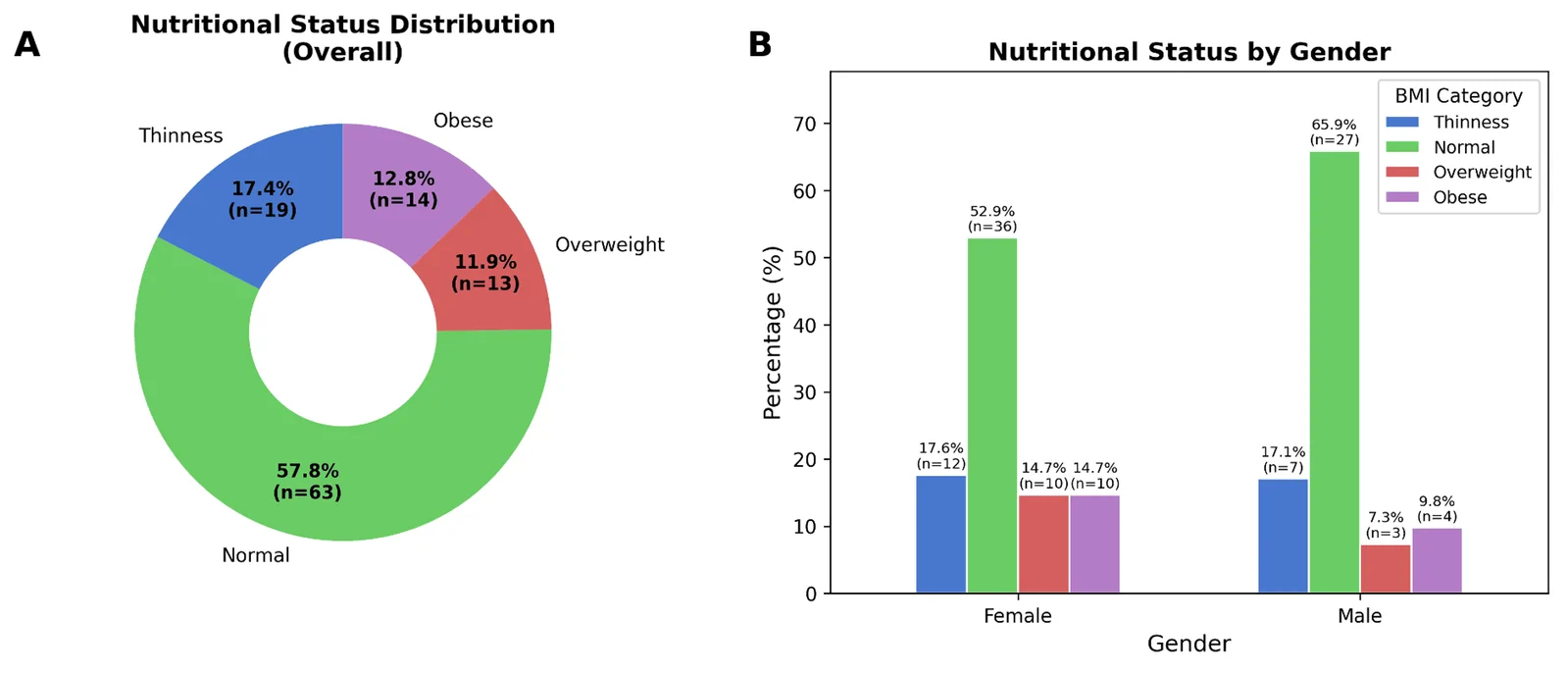
Distribution of nutritional status (n = 109). (A) Overall distribution by WHO 2007 BMI-for-age z-scores (% with n). (B) Distribution by gender.  All 109 enrolled adolescents had complete data and were analysed. Thinness: z < -2 SD; Normal: -2 to +1 SD; Overweight: > +1 to +2 SD; Obese: > +2 SD.

By WHO 2007 BMI-for-age z-scores, 57.8% of participants were normal, 17.4% thin, 11.9% overweight, and 12.8% obese; combined overweight/obesity was 24.8% (95% CI: 17.6%-33.6%). The concurrent thinness prevalence of 17.4% (95% CI: 11.5%-25.6%) alongside the overweight/obesity burden exemplifies the double burden of malnutrition. Neither the full four-category nutritional-status distribution nor the binary overweight/obesity outcome differed significantly by sex or age group: overweight/obesity affected 29.4% of females versus 17.1% of males, and 27.3% of early versus 23.7% of late adolescents (all p > 0.2). The complete cross-tabulation by sex and age group, with chi-square tests for both the four-category and the binary outcomes, is presented in Table 3; the overall category-wise prevalences are shown in Table 4 and Figure 1.

**Table 3:** Prevalence of overweight/obesity by sex and age group (n = 109). Values are within-group counts and row percentages (n (%)). *Chi-square test for the four-category nutritional-status distribution. The binary overweight/obese outcome was also non-significant for both sex (χ² = 1.480, df = 1, p = 0.224) and age group (χ² = 0.025, df = 1, p = 0.875). df = degrees of freedom.

Characteristic	Category	Thinness n (%)	Normal n (%)	Overweight n (%)	Obese n (%)	Overweight/obese n (%)	χ² (df); p*
Sex	Female (n = 68)	12 (17.6)	36 (52.9)	10 (14.7)	10 (14.7)	20 (29.4)	χ² = 2.401 (3); p = 0.493
	Male (n = 41)	7 (17.1)	27 (65.9)	3 (7.3)	4 (9.8)	7 (17.1)	
Age group	Early, 10-14 y (n = 33)	7 (21.2)	17 (51.5)	6 (18.2)	3 (9.1)	9 (27.3)	χ² = 2.783 (3); p = 0.426
	Late, 15-19 y (n = 76)	12 (15.8)	46 (60.5)	7 (9.2)	11 (14.5)	18 (23.7)	

**Table 4:** Prevalence of nutritional status by WHO 2007 BMI-for-age z-scores (n = 109). 95% CIs computed by the Wilson score method.

Nutritional status	n	%	95% CI (%)
Thinness (z < -2 SD)	19	17.4	11.5-25.6
Normal (z = -2 to +1 SD)	63	57.8	48.4-66.6
Overweight (z > +1 to +2 SD)	13	11.9	7.1-19.3
Obese (z > +2 SD)	14	12.8	7.8-20.4
Combined overweight + obese	27	24.8	17.6-33.6

Composite Dietary Antioxidant Index by sex and age

**Figure 2:**
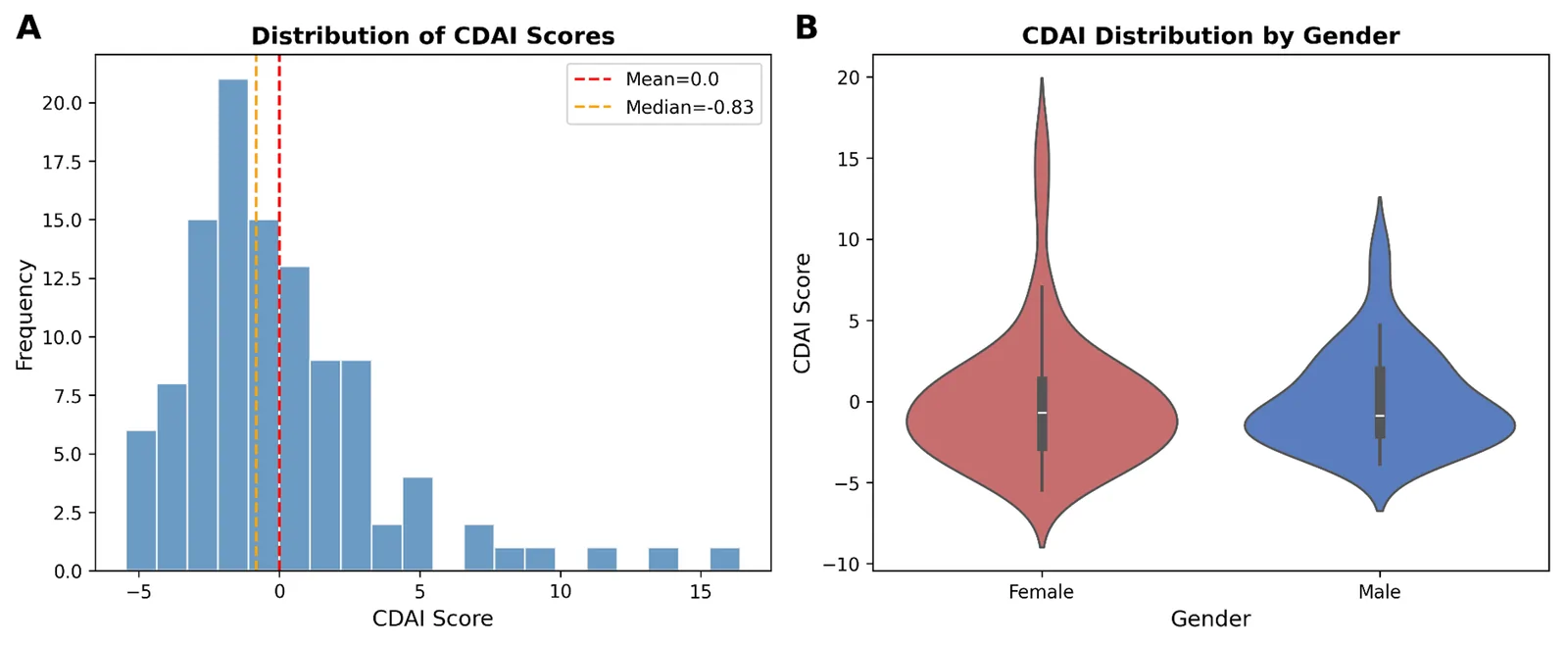
Distribution of CDAI scores. (A) Histogram with mean (red dashed) and median (orange dashed). (B) Violin plot of CDAI by gender; the inner box shows the interquartile range and median. CDAI = Composite Dietary Antioxidant Index.

The CDAI averaged 0.00 ± 3.72 (median: -0.83), the negative median reflecting a right-skewed distribution driven by a minority with high intakes. CDAI did not differ by sex (males: 0.09 ± 3.03; females: -0.05 ± 4.10; Mann-Whitney U, p = 0.489). By age group, late adolescents had a higher CDAI (mean: 0.35; median: -0.19) than early adolescents (mean: -0.81; median: -1.48), a difference that was statistically significant (Mann-Whitney U, p = 0.036) and consistent with somewhat greater dietary diversity in older adolescents. Summary statistics by sex and age group are presented in Table 5, and the CDAI distribution overall and by sex is shown in Figure 2.

**Table 5:** Composite Dietary Antioxidant Index by sex and age group (n = 109). Late adolescents had a significantly higher CDAI than early adolescents (p = 0.036). CDAI = Composite Dietary Antioxidant Index; SD = standard deviation; IQR = interquartile range.

Characteristic	Category	n	Mean ± SD	Median (IQR)	Test; p
Overall		109	0.00 ± 3.72	-0.83 (-2.32 to 1.53)	—
Sex	Female	68	-0.05 ± 4.10	-0.70 (-2.74 to 1.22)	Mann-Whitney U; p = 0.489
	Male	41	0.09 ± 3.03	-0.88 (-1.97 to 1.84)	
Age group	Early adolescence (10-14 y)	33	-0.81 ± 3.74	-1.48 (-3.04 to -0.25)	Mann-Whitney U; p = 0.036
	Late adolescence (15-19 y)	76	0.35 ± 3.68	-0.19 (-2.06 to 1.83)	

Association of the macronutrients, CDAI and its components with nutritional status

**Figure 3:**
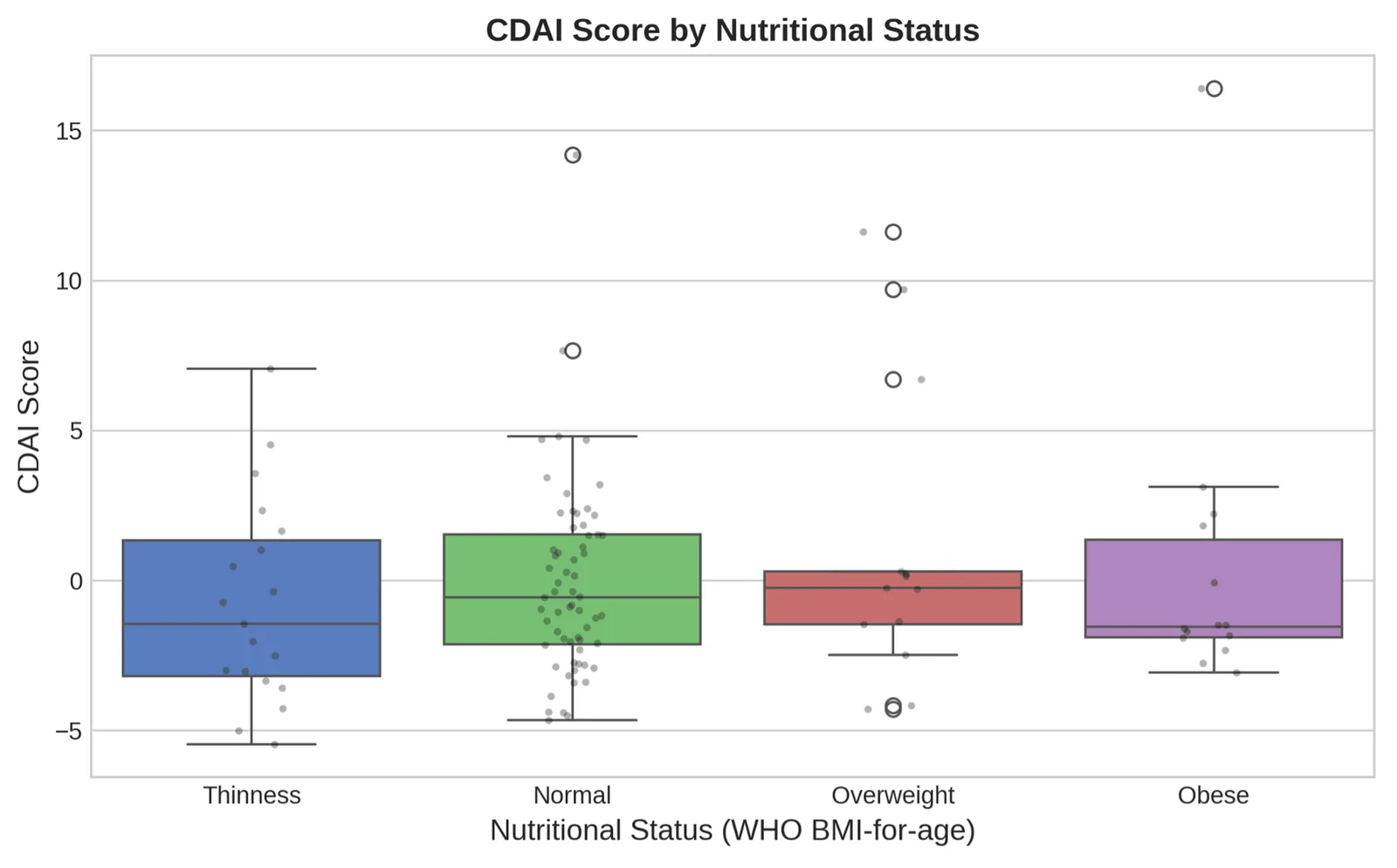
CDAI by nutritional-status category (thinness n = 19, normal n = 63, overweight n = 13, obese n = 14). Boxes show median and IQR; whiskers 1.5×IQR. Medians were comparable and distributions overlapped (Kruskal-Wallis H = 1.326, df = 3, p = 0.723). CDAI = Composite Dietary Antioxidant Index; IQR = interquartile range.

**Figure 4:**
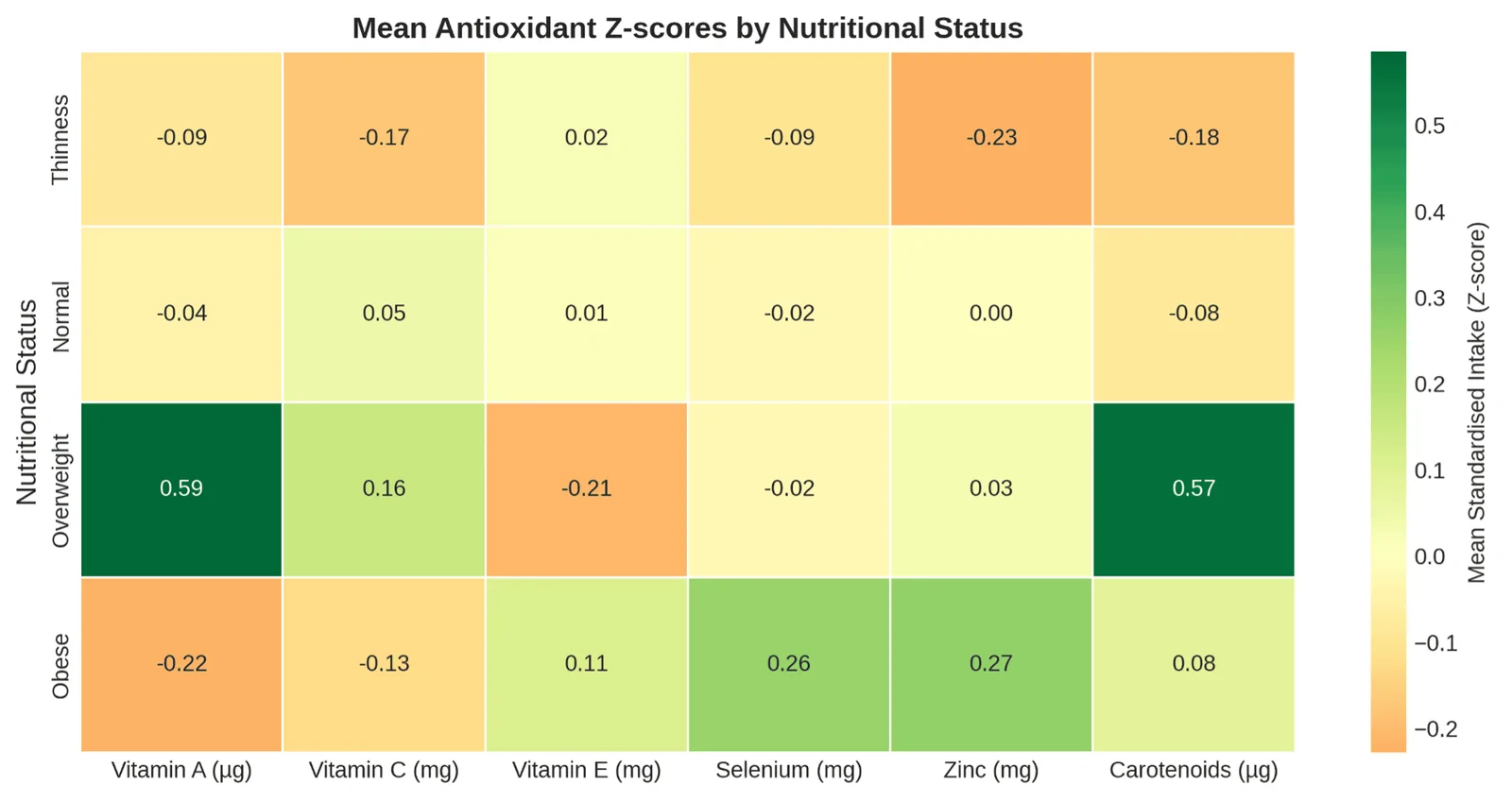
Heatmap of mean standardised (z-score) intakes of the six antioxidant components by nutritional status. Values fluctuate around zero with no consistent gradient; no between-group difference was significant (all p > 0.05).

**Figure 5:**
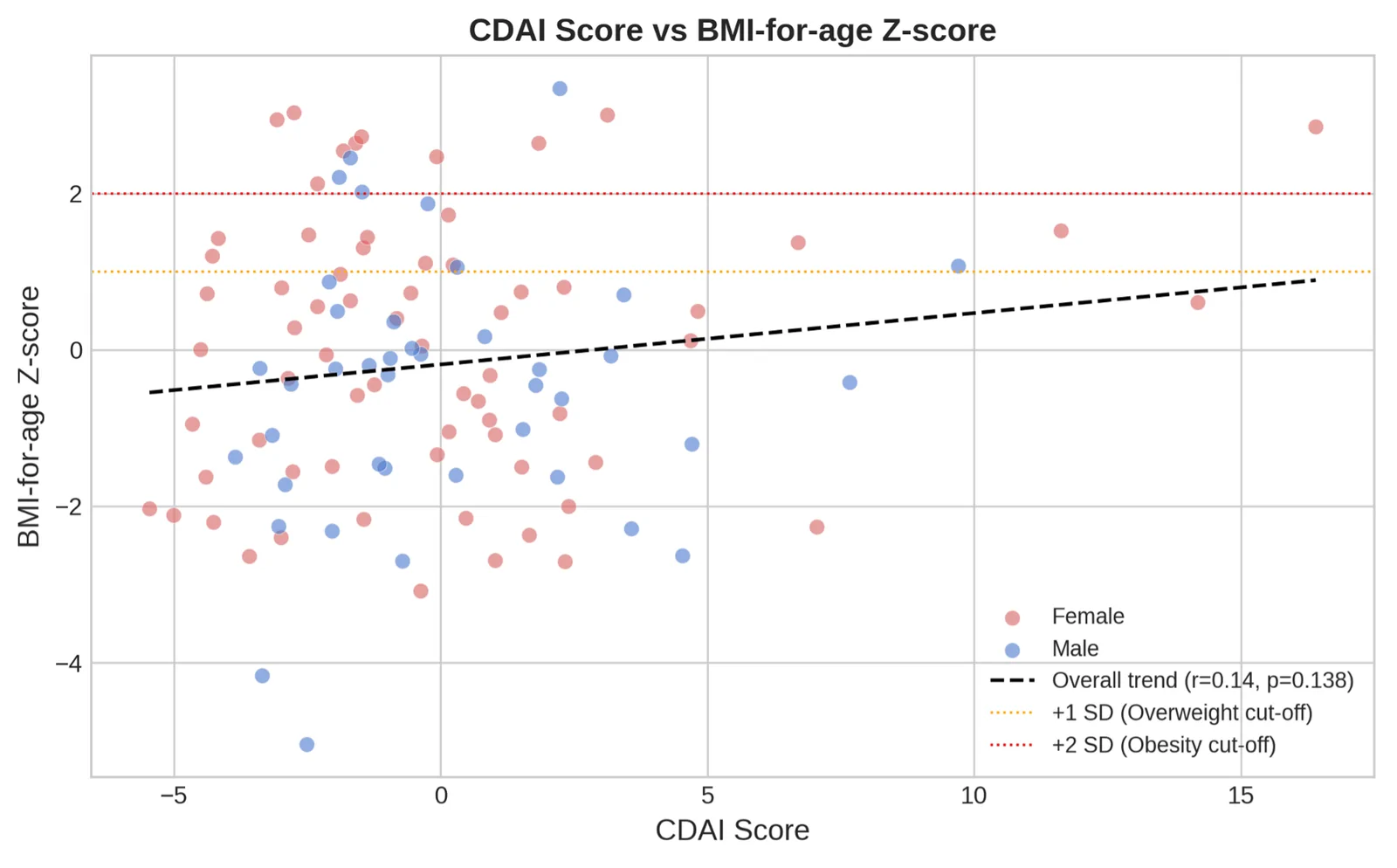
Scatter plot of CDAI versus BMI-for-age z-score (n = 109), colour-coded by sex. Dotted lines mark the WHO 2007 overweight (+1 SD) and obesity (+2 SD) cut-offs. No meaningful linear relationship was observed (Spearman ρ = 0.065, p = 0.505). CDAI = Composite Dietary Antioxidant Index.

CDAI did not differ significantly across the four nutritional-status categories (Kruskal-Wallis H = 1.326, df = 3, p = 0.723), and this held for every individual antioxidant component (all p > 0.05; Table 6). The combined overweight/obese group did not differ from the normal/thin group (Mann-Whitney U, p = 0.755). The apparently high mean vitamin A intake in the overweight group (283.8 µg) is an artefact of a single extreme value (3,140.6 µg); excluding it, the group mean falls to 45.8 µg, comparable to other categories, and the group median (26.6 µg) confirms no genuine gradient. When examined by CDAI tertile, overweight/obesity prevalence was non-monotonic (lowest tertile: 19.4%, middle: 36.1%, highest: 18.9%), providing no evidence of a dose-response relationship. In contrast to the antioxidant measures, carbohydrate intake differed significantly across nutritional-status categories (Kruskal-Wallis H = 8.583, df = 3, p = 0.035), being highest in the obese group (mean = 217.8 g/day); it was also higher in the combined overweight/obese group than in the normal/thin group (median = 198 vs 174 g/day; Mann-Whitney U, p = 0.032). Total energy (p = 0.432), protein (p = 0.870), and fat (p = 0.639) intakes did not differ across categories. Distributions by category are shown in Figures 3-5.

**Table 6:** Macronutrients, CDAI, and antioxidant components by nutritional status (n = 109). Data are mean ± SD. *Kruskal-Wallis test (df = 3); H = test statistic. Carbohydrate intake differed significantly across categories (p = 0.035), being highest in the obese group (shaded row). The overweight-group vitamin A mean is inflated by one extreme value (3,140.6 µg); the group median is 26.6 µg. CDAI = Composite Dietary Antioxidant Index.

Variable	Normal (n = 63)	Overweight (n = 13)	Obese (n = 14)	Thinness (n = 19)	H (df = 3)	p-value*
Energy (kcal)	1,292 ± 384	1,264 ± 335	1,430 ± 396	1,207 ± 268	2.746	0.432
Carbohydrate (g)	181.2 ± 54.3	183.8 ± 48.7	217.8 ± 50.8	169.1 ± 39.0	8.583	0.035
Protein (g)	40.0 ± 17.6	38.3 ± 15.0	42.7 ± 24.2	36.0 ± 14.6	0.712	0.870
Fat (g)	43.9 ± 16.4	39.6 ± 12.6	40.5 ± 14.2	40.0 ± 13.4	1.691	0.639
CDAI score	-0.09 ± 3.17	1.10 ± 5.04	0.38 ± 4.99	-0.75 ± 3.42	1.326	0.723
Vitamin A (µg)	72.9 ± 198.7	283.8 ± 859.9	14.3 ± 22.8	57.3 ± 96.6	3.975	0.264
Vitamin C (mg)	30.2 ± 29.7	33.1 ± 26.2	25.7 ± 19.9	24.4 ± 17.5	1.197	0.754
Vitamin E (mg)	1.39 ± 1.11	1.07 ± 0.64	1.53 ± 2.80	1.40 ± 1.51	1.753	0.625
Selenium (µg)	30.9 ± 24.9	31.0 ± 22.2	40.0 ± 58.6	28.7 ± 32.3	1.364	0.714
Zinc (mg)	4.77 ± 2.31	4.83 ± 2.21	5.34 ± 2.06	4.27 ± 1.64	1.398	0.706
Carotenoids (µg)	4,405 ± 2,391	6,677 ± 7,022	4,988 ± 3,145	4,062 ± 3,278	2.058	0.560

Correlation analysis

**Figure 6:**
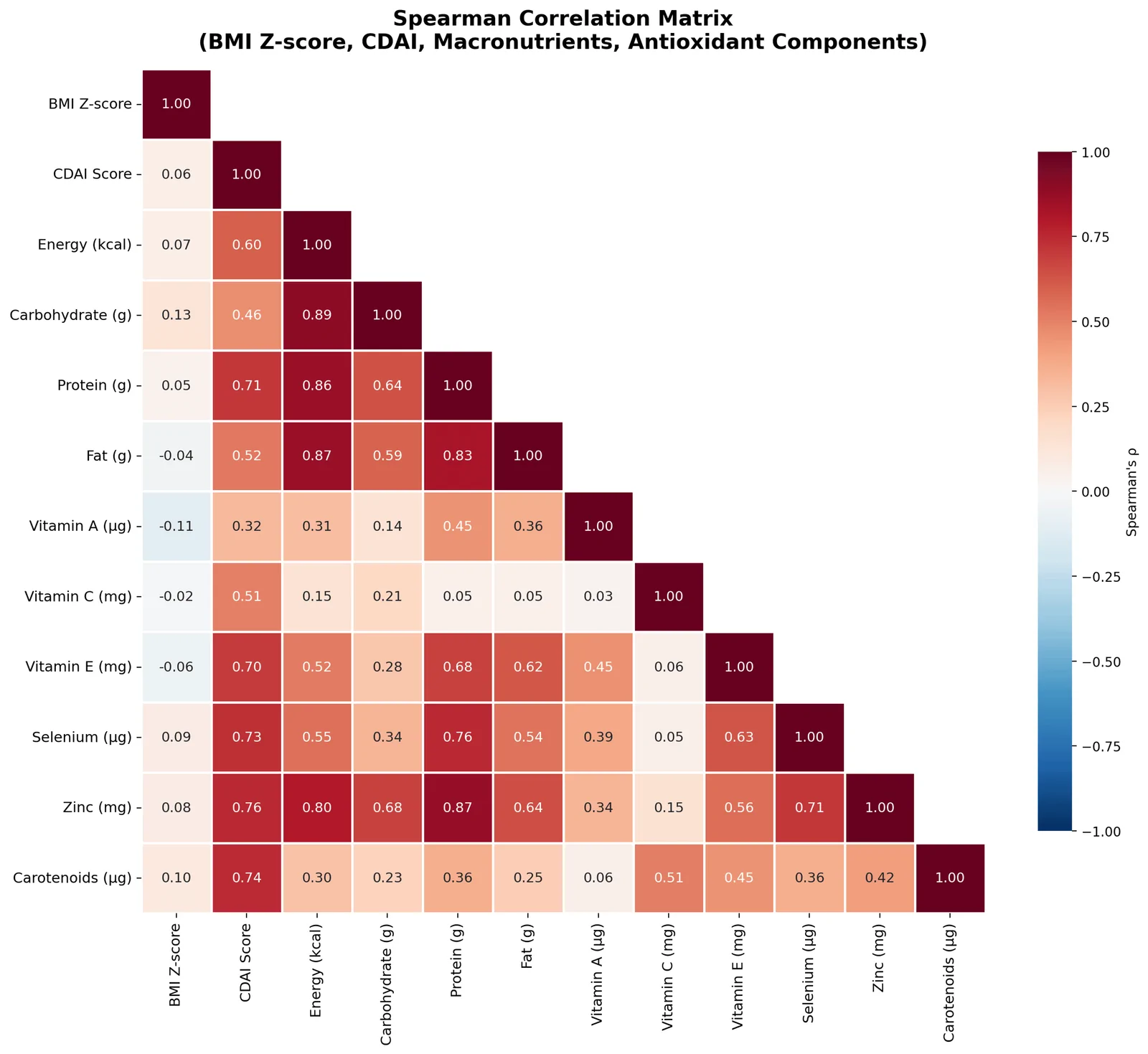
Spearman correlation matrix of BMI-for-age z-score, CDAI, macronutrients (energy, carbohydrate, protein, fat), and the individual antioxidant components. CDAI correlates strongly with its antioxidant components by construction (ρ = 0.32-0.76), and the macronutrients are strongly inter-correlated as expected for compositional intakes (energy with carbohydrate, protein, and fat, ρ = 0.86-0.89). BMI-for-age z-score shows only weak, non-significant correlations with all dietary variables (ρ = -0.11 to 0.13; all p > 0.05). CDAI = Composite Dietary Antioxidant Index.

Spearman correlation showed no relationship between CDAI and BMI-for-age z-score (ρ = 0.065, p = 0.505), and none of the individual components was significantly correlated with BMI-for-age z-score (all p > 0.05; Table 7, Figure 6).

**Table 7:** Spearman correlations with BMI-for-age z-score (n = 109). All correlations tested against BMI-for-age z-score. No correlation reached statistical significance (all p > 0.05). CDAI = Composite Dietary Antioxidant Index.

Variable	Spearman ρ	p-value
CDAI score	0.065	0.505
Energy (kcal)	0.067	0.486
Carbohydrate (g)	0.133	0.167
Protein (g)	0.045	0.641
Fat (g)	-0.046	0.634
Vitamin A (µg)	-0.105	0.276
Vitamin C (mg)	-0.019	0.843
Vitamin E (mg)	-0.055	0.569
Selenium (µg)	0.085	0.377
Zinc (mg)	0.082	0.395
Carotenoids (µg)	0.095	0.325

Predictors of overweight/obesity: logistic regression

In unadjusted logistic regression (Model 1), the CDAI was not associated with overweight/obesity (OR = 1.07 per 1-unit increase; 95% CI: 0.96-1.19; p = 0.246). Adjustment for age, sex, and physical activity (Model 2) left the estimate essentially unchanged (OR = 1.08; 95% CI: 0.96-1.21; p = 0.192). Because socioeconomic status plausibly confounds both food access and adiposity, Model 3 additionally adjusted for father's education and the PCA-derived household wealth index (in tertiles); the CDAI estimate remained non-significant and virtually identical (OR = 1.08; 95% CI: 0.96-1.22; p = 0.181), and neither socioeconomic indicator was significant (father's education OR = 0.93, p = 0.572; wealth index per tertile OR = 1.19, p = 0.583). Overweight/obesity prevalence was essentially flat across wealth tertiles (poorer 25.0%, middle 25.0%, richer 24.3%). A sensitivity analysis substituting the National Family Health Survey 2 (NFHS-2) Standard of Living Index for the wealth index yielded materially identical estimates. Variance inflation factors were low for all predictors (all VIF < 1.2), indicating no problematic multicollinearity. Full results are in Table 8.

**Table 8:** Binary logistic regression for overweight/obesity. *Reference category: Low physical activity. Model 2 adjusts for age, sex, and physical activity; Model 3 additionally adjusts for socioeconomic status (father's education and the PCA-derived household wealth index, in study-sample tertiles). All VIF < 1.2. McFadden pseudo-R² ≤ 0.06. OR = odds ratio; CI = confidence interval; SES = socioeconomic status; PCA = principal components analysis, CDAI = Composite Dietary Antioxidant Index, VIF: variance inflation factors.

Model	Predictor	OR	95% CI	p
Model 1 (unadjusted; n = 109)	CDAI (per 1 unit)	1.07	0.96-1.19	0.246
Model 2 (adjusted; n = 109)	CDAI (per 1 unit)	1.08	0.96-1.21	0.192
	Age (years)	0.84	0.65-1.07	0.151
	Sex (male vs female)	0.42	0.15-1.20	0.106
	Physical activity - Medium*	1.73	0.58-5.13	0.322
	Physical activity - High*	1.34	0.20-9.16	0.764
Model 3 (+ SES; n = 106)	CDAI (per 1 unit)	1.08	0.96-1.22	0.181
	Age (years)	0.83	0.65-1.07	0.156
	Sex (male vs female)	0.45	0.16-1.28	0.134
	Physical activity - Medium*	1.84	0.61-5.55	0.280
	Physical activity - High*	1.35	0.20-9.40	0.759
	Father's education (per level)	0.93	0.73-1.19	0.572
	Wealth index (per tertile)	1.19	0.64-2.22	0.583

## Discussion

In this facility-based sample of 109 adolescents attending an adolescent health clinic in Guntur, Andhra Pradesh, we found a combined overweight/obesity prevalence of 24.8% coexisting with 17.4% thinness, a clear double burden of malnutrition, while dietary antioxidant intake, measured by the CDAI, was not associated with nutritional status in any analysis, including after adjustment for socioeconomic status. Late adolescents had a modestly higher CDAI than early adolescents, but antioxidant intake did not track with adiposity.

Our combined overweight/obesity estimate (24.8%) is substantially higher than the national CNNS figure for adolescents (overweight 4.8% and obesity 1.1%, i.e., ~5.9% combined) [3], but this is expected: the CNNS is a community sample, whereas ours is a clinic-attending population in which adolescents with weight-related concerns may be over-represented, and the catchment is a nutritionally transitioning peri-urban district. The estimate lies within the wide range reported by a scoping review of Indian adolescent studies (overweight = 1.25%-35.8%; obesity = 0.3%-24.6%) [4] and is consistent with South Indian reports of higher-than-national prevalence attributed to nutrition transition, sedentary behaviour, and dietary change [17]. The concurrent 17.4% thinness prevalence mirrors the double burden documented across South Asian adolescents [18], and the direction of our age-group finding (numerically higher overweight/obesity in early adolescents) is concordant with the CNNS observation that early adolescents have higher odds of overweight than late adolescents [3].

The median CDAI of -0.83 indicates that most adolescents had below-(sample)-average antioxidant intakes, with the distribution right-skewed by a minority of high consumers. The high proportion of zero vitamin A intakes (31.2%) is notable and consistent with limited consumption of green leafy vegetables, liver, and dairy in lower-income households and with reported nutrient-intake patterns of Indian adolescents [19], although, as noted below, a single 24-hour recall overstates zero-intake days for such episodically consumed foods.

The absence of an association between the CDAI and adiposity contrasts with some prior reports. A study in Iranian adolescent boys described an inverse association between a dietary antioxidant index and BMI in Iranian adolescent boys (OR: 0.85, 95% CI: 0.76-0.96) [20], and Chen and Shi (2025) found higher composite antioxidant intake associated with lower overweight/obesity risk in National Health and Nutrition Examination Survey (NHANES) 2011-2016 [21]. Several factors may explain the divergence. Most importantly, our study was underpowered for this aim: with n = 109, the minimum correlation detectable at 80% power was |ρ| ≈ 0.27, and the achieved power to detect the observed ρ = 0.065 was only ~10%; likewise, the smallest odds ratio detectable per 1-SD of CDAI was ≈ 1.86. A true weak association could therefore have been missed. In addition, a single-day recall imperfectly captures habitual intake; the predominantly low-income sample may have diets constrained by availability rather than preference. Notably, in this setting, adiposity appeared to relate more to macronutrient than to antioxidant intake: carbohydrate intake was significantly higher in overweight/obese adolescents (highest in the obese group; across-category p = 0.035), whereas total energy, protein, and fat intakes did not differ significantly by nutritional status. These data provide preliminary support for the possibility that higher carbohydrate intake contributes to obesity in this population; however, the cross-sectional design and single 24-hour dietary recall preclude any firm or causal conclusions.

Mechanistically, oxidative stress, quantifiable by lipid-peroxidation markers such as TBARS, is elevated in obese adolescents, and dietary antioxidants can theoretically neutralise excess ROS [8,13]. However, whether higher antioxidant intake reduces adiposity itself, as opposed to attenuating its metabolic complications, remains uncertain, particularly where energy balance is the dominant driver. Because our design is cross-sectional, we make no causal claims in either direction. Physical activity was not associated with overweight/obesity (χ² = 1.207, p = 0.547), but with 89.9% of participants in the low or medium categories, limited variability constrained our ability to detect an effect and may itself represent residual confounding.

Strengths and limitations

Strengths include standardised WHO 2007 BMI-for-age classification; a reproducible CDAI derivation with an explicit formula; matched statistical testing; and multivariable modelling that included a socioeconomic status adjustment and collinearity diagnostics. To our knowledge, this provides among the first CDAI data for adolescents from this region of India. Limitations include the cross-sectional design, which precludes causal or temporal inference; reliance on a single 24-hour dietary recall, which may not represent habitual intake, was not balanced for weekday/weekend, and is subject to recall and social-desirability bias, particularly inflating zero-intake days for episodically consumed, vitamin A-rich foods; the absence of biochemical oxidative-stress markers (e.g., serum TBARS, total antioxidant capacity); the modest sample size, which left the study underpowered for the correlation/regression aim as quantified above; and selection bias inherent to a clinic-attending sample, which limits generalisability to the wider adolescent community.

## Conclusions

Among adolescents attending an adolescent health clinic in Guntur, Andhra Pradesh, a high combined overweight/obesity prevalence of 24.8% (27/109) coexisted with substantial thinness of 17.4% (19/109), reflecting a double burden of malnutrition within a single facility-based population. Dietary antioxidant consumption was poor: the median CDAI was below the sample average and nearly one-third of adolescents, 31.2% (34/109), consumed no vitamin A on the day of recall. Nevertheless, dietary antioxidant intake as measured by the CDAI was not associated with nutritional status in any analysis, including after adjustment for socioeconomic status, an inference tempered by the modest sample size, which afforded power to detect only moderate associations.

These findings indicate that adolescent nutrition programmes in this setting should address under- and overnutrition together rather than either pole in isolation, through a coordinated package of measures. Independent of the null CDAI-adiposity association, the high prevalence of negligible vitamin A intake justifies promoting access to and consumption of vitamin A- and carotenoid-rich foods (such as green leafy vegetables, orange and yellow fruits and vegetables, dairy, and eggs) in preference to antioxidant supplementation. This should be complemented by school- and family-based counselling on diverse, balanced diets; measures to strengthen physical activity and reduce sedentary behaviour; and routine BMI-for-age screening with nutrition education embedded within adolescent health services and existing national platforms such as the Rashtriya Kishor Swasthya Karyakram (RKSK), the Mid-Day Meal scheme, supported by staple-food fortification to help close micronutrient gaps. Finally, larger, prospective studies employing multiple 24-hour dietary recalls, energy adjustment of nutrient intakes, community-representative sampling, and biochemical markers of oxidative stress (for example, serum TBARS and total antioxidant capacity) are needed to clarify whether, and how, dietary antioxidant intake relates to adiposity in Indian adolescents.

## Appendices

Appendix 1 

**Table 9:** Structured questionnaire for assessment of dietary intake, anthropometry, and sociodemographic profile of adolescents.

Part I General Information							
1.	Unique ID of participants						
2.	Name						
3.	Age in year		4. Date of birth if available				
5.	Gender	1. Male		2. Female			
6.	Class studying						
7.	Location/address of the home?						
8.	Father's Occupation?	1. Farmer	2. Coolie or Daily Labour		3. Private Job	4. Government Job	5. Small Business
		6. Fisheries	7. Retired		8. Not working	9. Others (Specify)	
9.	Mother's Occupation?	1. Farmer	2. Coolie or Daily Labour		3. Private Job	4. Government Job	5. Small Business
		6. Fisheries	7. Retired		8. Not working	9. Others (Specify)	
10.	Father's education level?	1. Illiterate	2. Below the primary		3. Primary	4. Middle	5. High School
		6. Intermediate	7. Graduate		8. Postgraduate	9. Do not know	
11.	Mother's education level?	1. Illiterate	2. Below the primary		3. Primary	4. Middle	5. High School
		6. Intermediate	7. Graduate		8. Postgraduate	9. Do not know	
12.	Number of members in the family?		13. How many male siblings do you have?			14. How many female siblings do you have?	
15.	Order of the child in siblings?		16. Number of rooms in your house?				
17.	House type*	4 pucca	2 Semi Pucca		0 Katcha		
18.	Toilet Facility	4 Own Flush	2 Shared Flush		1 Public pit	0 No facility/open place	
19.	Main Fuel for cooking	3 Petroleum gas	1 Kerosene		0 Wood		
20.	Source of drinking water	2 Pipe/hand pump/well	1 Public tap		0 Others		
21.	Separate room for cooking	1 Yes	0 No				
22.	Ownership of house	1 Yes	0 No				
23.	Ownership of agricultural land	4 >5 acres	3 2-5 acres		2 <2 acres	0 No land	
24.	Livestock ownership	2 Yes	0 No				
25.	Ownership of durable goods	4 Car/tractor	3 Moped/scooter/motorcycle/telephone/refrigerator/colour tv		2 Bicycle/Electric fan/radio/transistor/tape recorder/sewing machine/black & white TV/water pump/bullock cart/thresher	1 Mattress/bed/pressure cooker/mixer/grinder/chair/cot/table/clock/watch	
26	Physical Activity	Daily Physical Activity	0-2		03 May	06 Jul	
		On how many days were you physically active for at least 60 minutes ?					
		Physical Activity at School	0-2		03-Apr	5+	
		On how many days did you participate in sports, games, or active play at school ?					
		Physical Activity After School/Weekends	0-2		03-Apr	5+	
		On how many days did you engage in sports, cycling, walking, or active play outside school hours ?					
27	Tobacco use	Tobacco product used ……………………..					
		Quantity……………..Frequency………………….. Years of use ……………….					
		If ex tobacco user stopped since (yr) ………………………					
28	Alcohol use	Alcohol product used ……………………..					
		Quantity……………… Frequency…………………..Years of use ……………..					
		If ex alcoholic , stopped since (yr) ……………………………					
29	Are you a known case of any disease : (specify) ………………………………………………						
	Hypertension/Diabetes/Hypothyroid/Hyperthyroid/Anemia/Asthma/COPD/ others.						
	( specify) ………………………………………………….						
	24 hr Dietary Recall						
	Sl no.	Time	Food item taken	Ingredients of food item	Quantity of food item		
